# Cutaneous Neuroimmune Interactions of TSLP and TRPV4 Play Pivotal Roles in Dry Skin-Induced Pruritus

**DOI:** 10.3389/fimmu.2021.772941

**Published:** 2021-12-02

**Authors:** Wook-Joo Lee, Won-Sik Shim

**Affiliations:** ^1^ College of Pharmacy, Gachon University, Incheon, South Korea; ^2^ Gachon Institute of Pharmaceutical Sciences, Incheon, South Korea

**Keywords:** dry skin, TSLP, TRPV4, keratinocyte, sensory neuron, mast cell, pruritus

## Abstract

Dry skin is a symptom of skin barrier dysfunction that evokes pruritus; however, the cutaneous neuroimmune interactions underlying dry skin-induced pruritus remain unclear. Therefore, we aimed to elucidate the mechanisms underlying dry skin-induced pruritus. To this end, an acetone/ethanol/water (AEW)-induced mouse model of dry skin was used in this study. We observed that the production of thymic stromal lymphopoietin (TSLP) significantly increased in the keratinocytes of AEW mice. Importantly, treatment with an antagonist of transient receptor potential cation channel subfamily V member 4 (TRPV4), HC067047, ameliorated dry skin conditions in AEW mice. The symptoms of dry skin were significantly reduced in *Trpv4* knockout (KO) mice following treatment with AEW. The increase in the intracellular calcium levels by TSLP in the dorsal root ganglia (DRG) of *Trpv4* KO mice was also significantly attenuated. The spontaneous scratching bouts were significantly decreased in both the HC067047-treated and *Trpv4* KO AEW mice. Importantly, the TSLP-dependent release of tryptase from the mast cells was significantly reduced in both the HC067047-treated mice and *Trpv4* KO AEW mice. Notably, inhibition of the TSLP-induced signaling pathway in DRG selectively reduced the spontaneous scratching bouts in AEW mice. Overall, the results demonstrated that the cutaneous neuroimmune interactions of TSLP and TRPV4 play pivotal roles in dry skin-induced pruritus.

## Introduction

Dry skin refers to a condition in which the skin becomes scaly, rough, and occasionally cracked, and leads to barrier dysfunction. Along with these morphological changes, dry skin induces pruritus, a sensation that evokes a desire to scratch. Although several theories have been suggested (1), it remains difficult to fully understand the precise mechanisms by which dry skin conditions evoke pruritus. In this study, we therefore aimed to elucidate the cutaneous neuroimmune interactions between dry skin conditions and pruritus.

To this end, an acetone/ethanol/water (AEW)-induced murine model of dry skin was used in this study, as this is the most well-known animal model for studying dry skin-induced pruritus (2). Treatment with AEW induces typical dry skin conditions, including an increase in transepithelial water loss (TEWL) and scratching behavior. Specifically, we focused on the putative molecular interactions among various cells including keratinocytes, peripheral sensory neurons, and mast cells. In particular, the effects and alterations induced by thymic stromal lymphopoietin (TSLP), and transient receptor potential cation channel subfamily V member 4 (TRPV4) were comprehensively investigated in AEW-treated mice.

TSLP is a histamine-independent pruritogenic cytokine that plays an important role in the pathogenesis of atopic dermatitis (3). TSLP is primarily produced by keratinocytes, and it directly acts on the TSLP receptor (TSLPR), inducing scratching behaviors in mice (4).

TRPV4 is a non-selective cationic ion channel that is expressed in sensory neurons and keratinocytes. TRPV4 is activated by thermal (27–35°C), mechanical, and chemical stimuli, as well as by numerous inflammatory metabolites (5). It has been recently reported that TRPV4 contributes to dry skin-induced pruritis, mediated by serotonin receptors (6). However, the relationship between TSLP and TRPV4 in dry skin-induced pruritus remains to be investigated.

In this study, we therefore aimed to investigate the putative interactions between TSLP and TRPV4, specifically in the keratinocytes, peripheral sensory neurons, and mast cells, for elucidating the mechanisms underlying dry skin-induced pruritus.

## Materials and Methods

### Reagents

Mouse TSLP was purchased from ProSpec (catalog #CYT-1135, East Brunswick, NJ, USA). GSK1016790A (catalog #G0798), HC067047 (catalog #616521), carvacrol (catalog #282197), 2-Aminoethoxydiphenylborane (2-APB, catalog #100065), SLIGRL-NH_2_ (SLIGRL, catalog #S9317), FSLLRY-NH_2_ (FSLLRY, catalog #SML0714), HC030031 (catalog #H4415), capsazepine (catalog #C191), baicalein (catalog #465119), dispase II (catalog #D4693), 4-nitrophenyl N-acetyl-β-D-glucosaminide (pNAG, catalog #N9376), and toluidine blue (catalog #89640) were purchased from Sigma-Aldrich (St, Louis, Missouri, USA).

### Animals

All the protocols for the animal experiments were approved by the Institutional Animal Care and Use Committee of Gachon University (GIACUC-R2020002) and were performed in accordance with the Guidelines for the Care and Use of Laboratory Animals issued by Gachon University. Nine-week-old male ICR and C57BL/6 mice were purchased from Koatech (Pyeongtaek, Gyeonggi-do, Korea). For the generation of *Trpv4* homozygous knockout (KO) mice (*Trpv4* ^-/-^), breeding pairs of heterozygous B6.129X1-Trpv4<tm1Msz> mice were purchased from RIKEN (Wako, Saitama Prefecture, Japan) (7, 8). The homozygous *Trpv4* KO offspring were verified by genotyping using the polymerase chain reaction (PCR) protocol provided by RIKEN. All *Trpv4* ^-/-^ mice possessed a homogeneous deletion of TRPV4. Littermate mice with genotypes of *Trpv4* ^+/+^ and T*rpv4* ^+/-^ were used for comparison. All animals showed no noticeable behavioral changes during the study period. The PAR2 KO mice were kindly gifted by Prof. Wan Namkung of Yonsei University.

### AEW-Treated Mouse Model of Dry Skin

The back of the nape was shaved, and the mice were randomly assigned to either the AEW group or the control group (n = 6 per group). The nape of the mice in the AEW group was painted with acetone and diethyl ether (1:1) for 15 s, followed by treatment with water for 60 s. The treatment was repeated twice daily (at 10 AM and 5 PM) for 5 consecutive days, as previously described (2). The nape of the mice in the control group were painted with water instead of acetone/diethyl ether.

### Evaluation of Skin Condition

The TEWL was measured using the Dermalab Combo system (C40000.03-189, Cortex Technology, Hadsund, Denmark) from the dorsal skin of the mice. A probe placed on the skin was stabilized for approximately 30 s, and each of the measurements were performed in triplicate, and averaged. The measurements were performed at a room temperature of 21-22°C and a relative humidity of 50%–55%, on days 1, 3, and 5 following AEW treatment. Additionally, the skin lesion scores were calculated as the sum of the two scores of erythema (redness) and desquamation (scaling). The score was evaluated based on severity, from a scale of 0 (no lesion) to 4 (worst severity).

### Enzyme-Linked Immunosorbent Assay (ELISA)

The levels of TSLP and tryptase were measured using a mouse TSLP ELISA kit (catalog #MTLP00; R&D Systems, Minneapolis, MN, USA) and mouse tryptase ELISA kit (catalog #RD-TPS-Mu; Reddot Biotech Inc., Kelowna, BC, Canada), respectively. Additionally, the serum levels of mouse IL-13, IL-33, and TNF-α were detected using a MILLIPLEX ELISA kit (catalog #MCYTOMAG-70K; Millipore, Billerica, MA, USA).

### Cell Culture, cDNA Transfection and Real-Time Quantitative PCR (RT-qPCR)

HaCaT cells were cultured in Dulbecco’s modified Eagle’s medium (DMEM; Gibco ThermoFisher, NY, USA) supplemented with 10% fetal bovine serum (FBS, Gibco ThermoFisher, NY, USA) and 10,000 U/mL of penicillin/streptomycin (Gibco ThermoFisher, NY, USA). The cells were maintained at 37°C in an incubator with a humidified atmosphere of 5% CO_2_. The cells were transfected with human PAR2 (NM_005242), TRPV3 (NM_001258205), and TRPV4 (NM_001177428) cDNA using a FuGENE^®^ HD transfection kit (Promega, Madison, WI, USA), according to the manufacturer’s protocol. Further experimentation was performed after 24 h of transfection.

### Real-Time Quantitative PCR (RT-qPCR)

The total RNA was extracted using a Total RNA Extraction Kit (iNtRON, Gyeonggi-do, Korea). The synthesis of the first-strand cDNA was performed using a PrimerScript™ 1^st^ strand cDNA Synthesis Kit (TaKaRa, Shiga, Japan). RT-qPCR was performed using a CFX96 qPCR System (BIO-RAD, CA, USA) with TB Green^®^ Premix Ex Tag™ II (Tli RNaseH Plus) and ROX Plus (catalog #RR82LR; TaKaRa, Shiga, Japan). The following protocol was used for RT-qPCR: Denaturation at 95°C for 30 s, annealing at 55°C for 60 s, and fluorescence detection at 72°C for 60 s for 40 cycles. For data analysis, glyceraldehyde 3-phosphate dehydrogenase (GAPDH) was used as the internal control. The primer pairs were custom designed for detecting the target genes (**Table S1**).

### Primary Culture of Murine DRG Neurons

The primary culture of DRG neurons was performed as previously described (9). Briefly, the murine DRG neurons were collected and incubated in Neurobasal^®^ Medium (Gibco ThermoFisher, NY, USA) supplemented with 1 mg/mL collagenase type II (Worthington Biochemical, Lakewood, NJ, USA) for 40 min at 37°C in a shaking incubator (60 rpm). The DRG neurons were additionally incubated with 2.5 mg/mL trypsin (Gibco ThermoFisher, NY, USA) in HBSS (Gibco ThermoFisher, NY, USA) for 40 min. After 10 min of centrifugation at 30 × *g*, the cells were dissolved in Neurobasal^®^ Medium supplemented with 10% FBS, 50-100 ng/mL nerve growth factor (NGF; Invitrogen, Gaithersburg, MD, USA), and 100 U/mL Zell Shield (Minerva BioLabs, Berlin, Germany), and plated on a poly L-lysine-treated 8-well chamber (Lab-Tek, Naperville, IL, USA). The cells were subsequently incubated for 2 days at 37°C in an atmosphere of 5% CO_2_ and 95% relative humidity.

### Primary Culture of Murine Keratinocytes

The animals were sacrificed, and the tail skin was peeled off and cut into 2-3 cm-long pieces. The protocol for the isolation and culture of keratinocytes was based on existing literature (10). Briefly, the pieces of tail skin were first washed with phosphate-buffered saline (PBS) and incubated overnight with KC growth media (EpiLife^®^, Gibco ThermoFisher, NY, USA) containing 4 mg/mL dispase II in a rotator at 4°C. The following day, the pieces of tail skin along with the media were poured into a Petri dish and washed with PBS. The epidermis was lifted using forceps and transferred to a new Petri dish containing trypsin solution at room temperature. The epidermis was then gently agitated using a horizontal shaker at 30 rpm for 20 min. The epidermal tissues were vigorously shaken to disperse the keratinocytes into single cells. The cells were subsequently filtered through a 100 μm filter (Falcon, NY, USA), and centrifuged for 5 min at 180 × *g*. The supernatants were aspirated, and the pellets were gently resuspended in 1 mL ice-cold KC growth medium. The keratinocytes were seeded in 6-well plates at a density of 1 × 10^4^/cm^2^ in KC growth medium. The medium was replaced after 24 h of initial plating for removing the unattached cells, and the cells were cultured for three days.

### Peritoneal Mast Cell (PMC) Primary Culture

The primary culture of the PMCs was performed as previously described (11). Briefly, the mice were euthanized with CO_2_ prior to experimentation. The abdomen was opened, and 7 ml of ice-cold Roswell Park Memorial Institute (RPMI) 1640 medium (Gibco ThermoFisher, Gangnam, Korea) was added for detaching the PMCs from the peritoneum. The media containing the PMCs was centrifuged at 300 × *g* for 5 min, and the PMCs were seeded in 25T flasks with murine IL-3 (mIL-3, 10 ng/mL; Sigma-Aldrich, St, Louis, Missouri, USA) and murine stem cell factor (mSCF, 30 ng/mL; Peprotech, NJ, USA). After 48 h of incubation, the medium was replaced, and mIL-3 and mSCF were added again. The PMCs were cultured for 12 days until experimentation.

### β-Hexosaminidase Assay

The PMCs were transferred into 96-well V-bottomed plates and incubated for 1 h at 37°C in a 5% CO_2_ incubator. After 1 h of incubation, 5 ng/ml of mTSLP was added to the wells, and the PMCs were incubated for an additional 1 h at 37°C. The plate was subsequently centrifuged at 120 × *g* for 5 min at 4°C. Then, 195 µL of the supernatant was temporarily placed in a clean 96-well flat-bottomed plate and placed on ice until use. Additionally, 200 µL of lysis buffer (1% volume of Triton X-100 to 1 × NBS) was added to the cell pellets in 96-well V-bottomed plates and incubated for 5 min at room temperature. After 5 min, the pellets were resuspended by pipetting, and referred to as the lysate. In another clean flat-bottomed 96-well plate, 25 µL of 4 mM pNAG (pH 4.5 dissolved in 0.4 M citric acid) and 25 µL of either the supernatant or the lysate was added to the respective wells and incubated for 1 h. After 1 h of incubation, 150 µL of the stop solution (200 mM glycine, adjusted to pH 10.7) was added to each well to cease the reaction. The absorbance of the supernatant or the lysate were measured at 405 nm wavelength (A_supernatant_ or A_lysate_) using a microplate reader (Synergy H1 Hybrid Reader, BioTek, VT, USA). The percentage of β-hexosaminidase released, which serves as an indicator of mast cell degranulation, was calculated using the following equation:


$$\beta -hexosaminidase\;(\%)=\frac{A_{supernatant}}{A_{supernatant}+A_{lysate}}\times 100$$

where, β-hexosaminidase (%) represents the percentage of β-hexosaminidase released.

### Test for Scratching Behavior in Mice

Nine-week-old male ICR mice were purchased from Koatech (Pyeongtaek, Gyeonggi-do, Korea). Their spontaneous scratching behavior was recorded for up to 120 min. Under certain conditions, the mice were intraperitoneally pretreated with the appropriate compounds before 60 min of recording. The scratching bouts were counted by an experienced researcher. One bout of scratching was defined as the scratching movement of the hind limb near the injected area until it touched the floor.

### Calcium Imaging

Calcium imaging was performed with Fluo-3/AM (Invitrogen, Carlsbad, CA, USA) as described previously (9). Briefly, the intracellular calcium levels were determined using a fluorescence microscope (ECLIPSE Ti-U; Nikon, Tokyo, Japan; and Leica DMi8 inverted microscope; Leica Microsystems Ltd., Wetzlar, Germany) with the calcium-specific fluorescent dye, Fluo-3/AM (5 µM; Invitrogen, Carlsbad, CA, USA). Fluo-3/AM was mixed with 0.1% F127 in normal buffer solution (NBS: 140 mM NaCl, 5 mM KCl, 2 mM CaCl_2_/EDTA, 0.5 mM MgCl_2_, 10 mM glucose, and 5.5 mM HEPES, adjusted to pH 7.4), and loaded into the cells by incubating for 60 min at 37°C. After 60 min of incubation, the cells were washed twice with NBS and replaced with fresh NBS. Following treatment with the compounds, the alterations in the fluorescent images were recorded on a computer connected to the microscope. The excitation and emission wavelengths were 488 nm and 515 nm, respectively. The intracellular calcium levels were expressed as the F/F_0_ ratio, where F indicates the fluorescence intensity of the region of interest at a certain time point, and F_0_ indicates the initial fluorescence intensity at 0 s. Image analysis was performed using the ImageJ software (NIH), with custom-made scripts for semi-automatic cell counting, calculation of the F/F_0_ ratio, and image production.

### Immunostaining

The sections of murine skin were sliced using a cryostat, and the sections were fixed to the slides. The sections were washed with PBS and fixed with 4% paraformaldehyde for 10 min. Hydrogen peroxide (1%) was used for suppressing the endogenous peroxidase activity, and 0.3% Triton X-100 with 1% FBS was used for blocking. The samples were then incubated overnight at 4°C with a TRPV4 rabbit polyclonal antibody (catalog #ab39260; Abcam, Cambridge, UK) at a dilution of 1:200. The samples were washed with PBS the following day and incubated with a goat anti-rabbit IgG H&L secondary antibody (Alexa Fluor^®^ 488, catalog #ab150077; Abcam) for 2 h in the dark to avoid photobleaching. The primary and secondary antibodies were prepared in 0.3% Triton-X 100 containing 0.5% FBS. After washing, a DAPI staining solution freshly prepared in PBS (300 nM), was added to the samples, and incubated for 10 min. The samples were rinsed 2-3 times with PBS, mounted with Vectashield (Vector Laboratories, Burlingame, CA, USA), and covered with a coverslip. The samples were visualized, and the images were prepared using a Leica DMi8 inverted microscope (Leica Microsystem Ltd., Wetzlar, Germany).

### Hematoxylin & Eosin (H&E) Staining and Toluidine Blue Assay

The dorsal skin tissues of the mice were collected on the last day of the experiment, and subsequently fixed with 10% neutral-buffered formalin and embedded in paraffin. Then, 4 µm-thick skin sections were sliced and transferred to slides. The deparaffinized skin sections were stained with H&E or toluidine blue before examination at 100 × magnification. The stained skin sections were observed under a Nikon Eclipse 80i microscope (Tokyo, Japan) at 100 × magnification.

### Statistical Analyses

All the data are presented as the mean ± standard error of the mean (SEM), and the statistical analyses were performed using the GraphPad Prism software. Comparisons between the two groups were made using unpaired Student’s *t*-test, while comparisons among more than three groups were made by one-way analysis of variance (ANOVA) with Dunnett’s multiple comparison test or Tukey’s multiple comparison test. Fisher’s exact test was used for comparing the responsiveness of the DRG neurons. The statistical significance was considered at *p* < 0.05.

## Results

### AEW Mice Developed Dry Skin Conditions And Exhibited Increased TSLP Production

**Figure 1:**
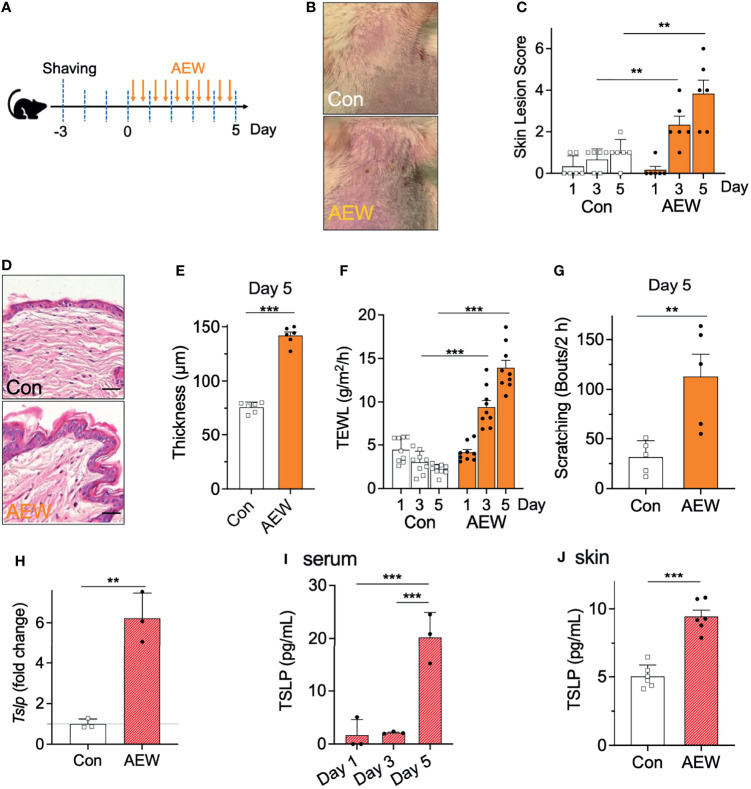
Development of dry skin symptoms and increased TSLP levels in AEW-treated mice. **(A)** Schematic illustration of the treatment protocol with AEW. **(B)** Images of the mouse skin, with or without AEW treatment. **(C)** A graph of the skin lesion scores of the control (n = 6) and AEW-treated mice (n = 6), as measured on different days. **(D)** Representative H&E-stained images of the skin tissues collected on day 5. **(E)** The thickness of the epidermis significantly increased on day 5 following AEW treatment (n = 6), compared to that of the control (n = 6). **(F)** The values of TEWL significantly increased on days 3 and 5 following AEW treatment (n = 9). **(G)** The spontaneous scratching bouts significantly increased in the AEW-treated mice (n = 5) on day 5, compared to those of the control (n = 5). **(H)** The transcriptional levels of *Tslp* significantly increased in the keratinocytes of AEW-treated mice. **(I)** The serum levels of TSLP significantly increased in the AEW-treated mice (n = 3) on day 5, compared to those of the control (n = 3). **(J)** The skin levels of TSLP increased on day 5 after treatment with AEW, compared to those of the control (n = 6). **p < 0.01, ***p < 0.001.

The AEW-induced murine model of dry skin was evaluated whether the model had developed the characteristic symptoms of dry skin. AEW was administered twice on a daily basis for five consecutive days (**Figure 1A**). On day 5, the AEW-treated mice exhibited typical dry skin-induced irritation (**Figure 1B**). The skin lesion scores were evaluated, and the scores of the AEW-treated mice were higher than those of the control mice from day 3 (**Figure 1C**). The skin of the AEW-treated mice exhibited typical epidermal thickening on day 5 (**Figures 1D, E**). Notably, the values of TEWL significantly increased in the AEW-treated mice from day 3, compared to those of the control (**Figure 1F**). On day 5, the spontaneous scratching behaviors of the AEW-treated mice significantly increased in comparison to those of the control (**Figure 1G** and **Figure S1**). Therefore, these data demonstrated that the AEW-treated mice successfully developed the representative characteristics of dry skin conditions.

We next investigated whether the levels of TSLP were altered in the AEW-treated mice. The results demonstrated that the transcriptional levels of *Tslp* in the keratinocytes were significantly increased in the AEW-treated mice (**Figure 1H**). Similarly, the serum levels of TSLP increased from day 5 in the AEW-treated mice, which were significantly higher than those of the control mice (**Figure 1I**). Furthermore, the levels of TSLP in the skin from the AEW-treated mice were also increased on day 5, and the levels were significantly different from those of the control mice (**Figure 1J**). In contrast, the levels of the other related cytokines such as IL-13 (**Figures S2A-C**) and TNF-α (**Figure S2G**) remained unaltered. The transcriptional level of IL-33 was increased in the skin of both AEW-treated ICR mice (**Figure S2D**) and *Trpv4* ^+/+^ mice (**Figure S2E**). In addition, we observed that the transcriptional levels of the positive control, aquaporin 3 (*AQP3)*, increased in the keratinocytes of AEW-treated mice (**Figure S2H**), which is in agreement with a previous report (12). Overall, these data suggest that the AEW-treated mice developed the characteristic dry skin conditions, and exhibited an increased production of TSLP.

### TRPV4 Partakes in the Elevated Production of TSLP in the Keratinocytes of AEW Mice

**Figure 2:**
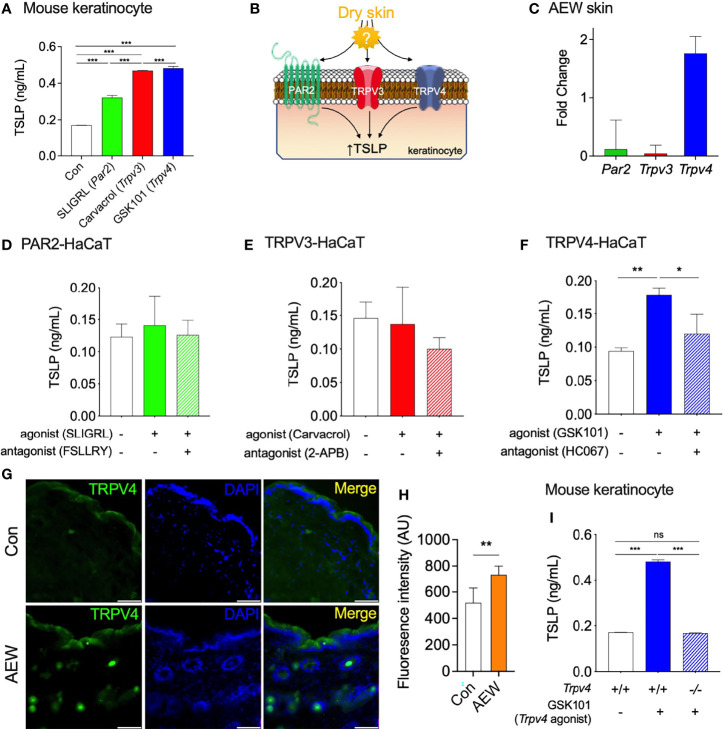
TRPV4 partakes in the elevated production of TSLP in dry skin conditions. **(A)** The production of TSLP in the murine keratinocytes was increased by SLIGRL (50 µM, a PAR2 agonist), carvacrol (100 µM, a TRPV3 agonist), and GSK101 (10 µM, a TRPV4 agonist). **(B)** A schematic illustration of the putative relationship between the elevated production of TSLP and proteins-of-interest (PAR2, TRPV3, and TRPV4) in dry skin conditions. **(C)** The transcriptional levels of *Trpv4* increased in the keratinocytes of AEW-treated mice, whereas those of *Par2* and *Trpv3* remained unaltered (n = 3). **(D)** The activation of PAR2 expressed in HaCaT cells did not alter the production of TSLP. PAR2 was activated by 50 µM SLIGRL (n = 3) and inhibited by 10 µM FSLLRY (n = 3). **(E)** The activation of TRPV3 expressed in HaCaT cells did not alter the production of TSLP. TRPV3 was activated by 100 µM carvacrol (n = 3) and inhibited by 100 µM of 2-APB (n = 3). **(F)** The activation of TRPV4 expressed in HaCaT cells induced the production of TSLP. TRPV4 was activated by 10 µM GSK101 (n = 3) and inhibited by 100 µM HC067047 (HC067; n = 3). **(G)** The immunostaining of TRPV4 in the skin tissues of AEW-treated mice revealed that the fluorescence intensity was higher than that of the control (**H**; AU: arbitrary unit). The scale bar indicates 50 µm. **(I)** The production of TSLP significantly increased in the keratinocytes of *Trpv4* ^+/+^ mice following treatment with 10 µM GSK101. However, the levels of TSLP in the keratinocytes of *Trpv4* ^-/-^ mice remained unaltered even after treatment with 10 µM GSK101. *p < 0.05, **p < 0.01, ***p < 0.001, n.s. not significant.

Previous studies have demonstrated that protease-activated receptor 2 (PAR2), TRPV3, and TRPV4, are involved in the production of TSLP in keratinocytes under dry skin conditions (6, 13, 14). To confirm these observations, the primary culture of mouse keratinocytes was treated with PAR2, TRPV3, and TRPV4 agonists, and the levels of TSLP were measured. As expected, the activation of these proteins increased the production of TSLP in mouse keratinocytes (**Figure 2A**), which verified the relationship between these proteins and TSLP. However, the mostly involved protein in the enhanced TSLP production under dry skin conditions remains to be identified (**Figure 2B**). Thus, we determined the transcriptional levels of three genes (*Par2*, *Trpv3*, and *Trpv4*) in the skin of AEW-treated and control mice. As depicted in **Figure 2C**, the transcriptional levels of *Trpv4* were markedly increased, whereas no noticeable changes in the transcriptional levels of *Par2* and *Trpv3*. This implied that TRPV4 could be a major player in the enhanced production of TSLP under dry skin conditions. In order to further verify the role of TRPV4, the genes were transiently transfected into the human keratinocyte-originated cell line (HaCaT) and the levels of TSLP were determined after treatment with respective agonists. As shown in **Figure 2D**, the activation of PAR2 had no effect on the production of TSLP in HaCaT cells. Similarly, the activation of TRPV3 did not produce any difference in the levels of TSLP (**Figure 2E**). On the other hand, the activation of TRPV4 by GSK1016790A (‘GSK101’) significantly increased the levels of TSLP in HaCaT cells, and the levels of TSLP reduced by co-treatment with the TRPV4 antagonist, HC067047 (‘HC067’; **Figure 2F**). Furthermore, the results of immunostaining studies on the skin tissues of AEW-treated mice revealed an increase in the expression of TRPV4, as depicted in **Figures 2G, H**. Importantly, GSK101 treatment did not increase the production of TSLP in keratinocytes of *Trpv4* KO (*Trpv4^-/-^*) mice (**Figure 2I**). Therefore, these results suggest that TRPV4 could play a crucial role in the enhanced production of TSLP under dry skin conditions.

### A TRPV4 Antagonist Alleviated the Dry Skin Symptoms of AEW-Treated Mice

**Figure 3:**
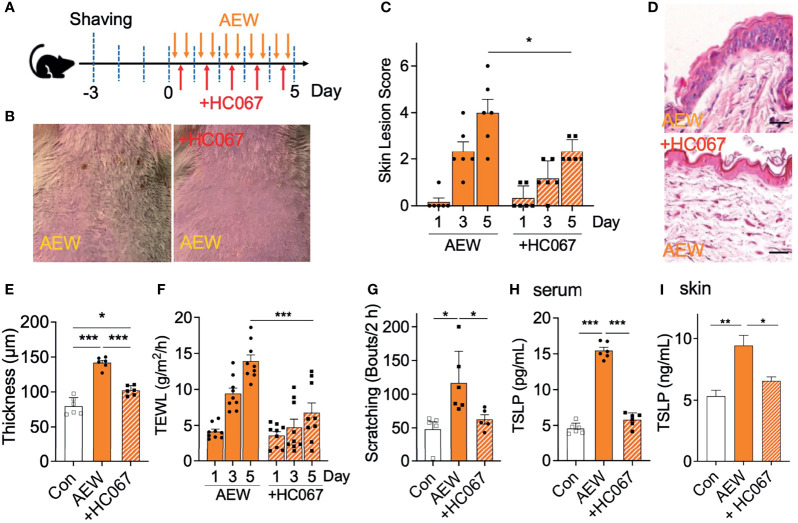
Treatment with a TRPV4 antagonist alleviated the dry skin symptoms of AEW-treated mice. **(A)** A schematic illustration depicting the administration of HC067047 (HC067) during treatment with AEW. **(B)** Pretreatment with HC067 alleviated the deterioration in the overall skin conditions following AEW treatment. **(C)** The skin lesion scores significantly reduced in the HC067-treated AEW mice on day 5 (n = 6). **(D)** Representative H&E-stained images of the skin lesions of AEW-treated mice with or without HC067 administration. **(E)** The thickness of the epidermis significantly decreased in the skin lesions of the HC067-treated AEW mice (n = 6), compared to that of the control (n = 6) **(F)** The values of TEWL significantly reduced in the HC067-treated AEW mice on day 5 (n = 9), compared to those of the control (n = 9). **(G)** The spontaneous scratching bouts were reduced in the HC067-treated AEW mice (n = 5), compared to those of the control (n = 6). **(H)** The serum levels of TSLP significantly decreased in the HC067-treated AEW mice (n = 6), compared to those of the control (n = 6). **(I)** TSLP in the skin significantly decreased in HC067-treated AEW mice (n = 6) compared to the AEW-treated mice (n = 6). *p < 0.05, **p < 0.01, ***p < 0.001.

We further examined whether the increased expression of TRPV4 in the skin of AEW-treated mice is related to the dry skin conditions. A selective TRPV4 antagonist, HC067047, was administered during treatment with AEW for 5 consecutive days (‘+HC067’; **Figure 3A**). As depicted in **Figure 3B**, the administration of HC067047 strongly protected the skin against AEW-induced dry skin. The skin lesion scores demonstrated that HC067047 had a protective effect on AEW-induced dry skin conditions (**Figure 3C**). HC067047 also suppressed the epidermal thickening induced by AEW (**Figures 3D, E**). The values of TEWL also decreased significantly on day 5, indicating that the dry skin condition was significantly ameliorated by HC067047 (**Figure 3F**). The spontaneous scratching behavior of AEW-treated mice was also greatly reduced by HC067047 (**Figure 3G**). Importantly, TSLP levels in the serum and the skin significantly decreased in the AEW-treated mice that received HC067047 (**Figures 3H, I**). Treatment of HC067047 alone did not evoke any changes on skin lesion scores, TEWL, skin thickness, and scratching bouts (**Figure S3**). Taken together, these results imply that TRPV4 plays an important role in the development of dry skin conditions.

### Reduced Dry Skin Symptoms and Decreased TSLP Levels in TRPV4-Deficient Mice Following AEW Treatment

**Figure 4:**
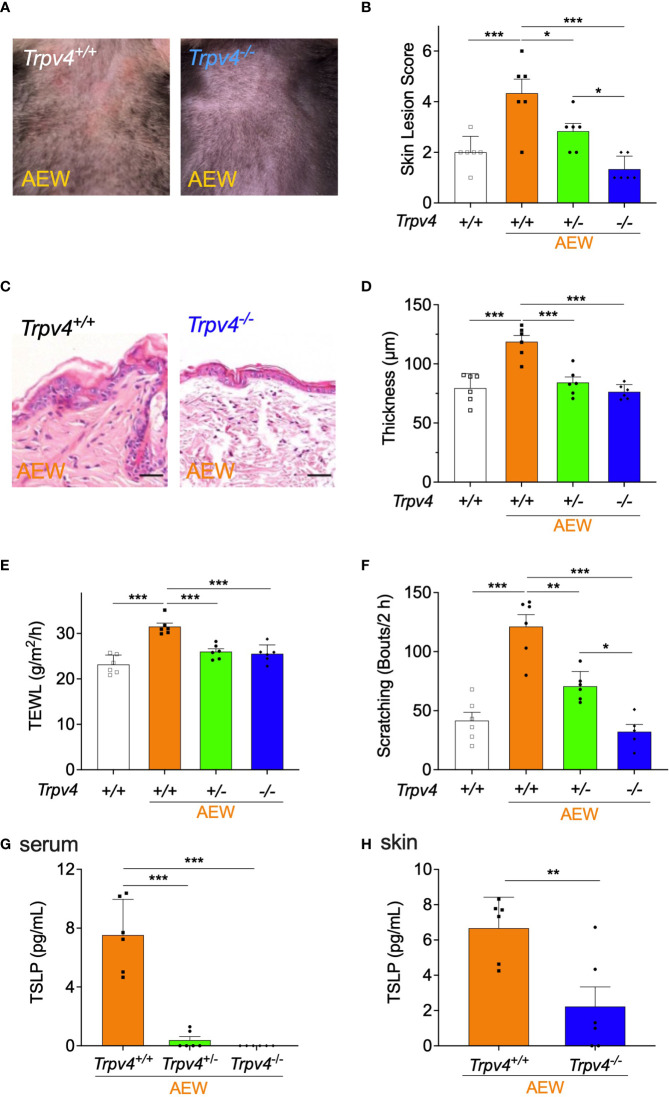
The symptoms of dry skin and reduction in TSLP levels were less pronounced in TRPV4-deficient mice following AEW treatment. **(A)** The skin of *Trpv4* ^-/-^ mice appeared less dry following AEW treatment. **(B)** On day 5 after treatment with AEW, the skin lesion scores gradually decreased in the following order: *Trpv4^+/+^* (n = 6) > *Trpv4^+/-^* (n = 6) > *Trpv4* ^-/-^ (n = 6). **(C)** Representative H&E-stained images of the skin tissues of *Trpv4^+/+^* and *Trpv4* ^-/-^ mice following AEW treatment. **(D)** The epidermal thickness significantly decreased in both AEW-treated *Trpv4^+/-^* mice (n = 6) and *Trpv4* ^-/-^ mice (n = 6). **(E)** The values of TEWL significantly reduced in the AEW-treated *Trpv4^+/-^* mice (n = 6) and *Trpv4* ^-/-^ mice (n = 6). **(F)** The spontaneous scratching bouts were reduced in AEW-treated *Trpv4^+/-^* mice (n = 6) and *Trpv4* ^-/-^ mice (n = 6). **(G)** The serum levels of TSLP significantly decreased in the AEW-treated *Trpv4^+/-^* mice (n = 6) and *Trpv4* ^-/-^ mice (n = 6). **(H)** The levels of TSLP in the skin significantly reduced in the AEW-treated *Trpv4* ^-/-^ mice (n = 6), compared to those of the control (n = 6). *p < 0.05, **p < 0.01, ***p < 0.001.

To further investigate the role of TRPV4 in the development of dry skin, *Trpv4*-deficient mice were used and treated with AEW. As depicted in **Figure 4A**, the skin lesions of homozygous *Trpv4* KO (*Trpv4^-/-^*) mice reduced on day 5 after AEW treatment. As shown in **Figure 4B**, the skin lesion scores of *Trpv4^-/-^* mice decreased significantly in comparison to those of *Trpv4* ^+/+^ mice, while the skin lesion scores of heterozygote KO (*Trpv4^+/-^*) mice decreased to a lesser extent. The transcriptional levels of *Trpv4* in the skin and the DRG are significantly reduced in *Trpv4* ^+/-^ mice compared to *Trpv4* ^+/+^ mice, and there was no detection of *Trpv4* in *Trpv4^-/-^* mice as expected (**Figures S4A, B**). The epidermal thickening of *Trpv4^-/-^* mice did not increase in comparison to that of the *Trpv4* ^+/+^ AEW-treated mice (**Figures 4C, D**), and a similar trend was also observed in *Trpv4^+/-^* mice (**Figure 4D**). Furthermore, the values of TEWL significantly decreased in both *Trpv4^+/-^* and *Trpv4^-/-^* mice (**Figure 4E**). The spontaneous scratching behaviors of both the *Trpv4^+/-^* and *Trpv4^-/-^* mice also decreased (**Figure 4F**). All mice showed no difference in skin lesion score, TEWL, skin thickness, and scratching bouts when there was no AEW treatment (**Figure S4C-F**). As *Trpv4^+/-^* and *Trpv4^-/-^* mice belong to the C57BL/6 strain, the spontaneous scratching behaviors of the C57BL/6 and ICR strains were compared after AEW treatment. The results demonstrated that there were no differences in the scratching behaviors of the two strains (**Figure S5**). Importantly, the TSLP was barely detected in the serum samples of *Trpv4^+/-^* and *Trpv4^-/-^* mice (**Figure 4G**). Furthermore, the levels of TSLP in the skin decreased significantly in *Trpv4^-/-^* mice (**Figure 4H**). Overall, these data suggest that TRPV4 and TSLP play a crucial role in the development of AEW-induced dry skin conditions in mice.

### Sensory Neurons of AEW-Treated Mice Showed Increased Responses to TSLP in a TRPV4-Dependent Manner

**Figure 5:**
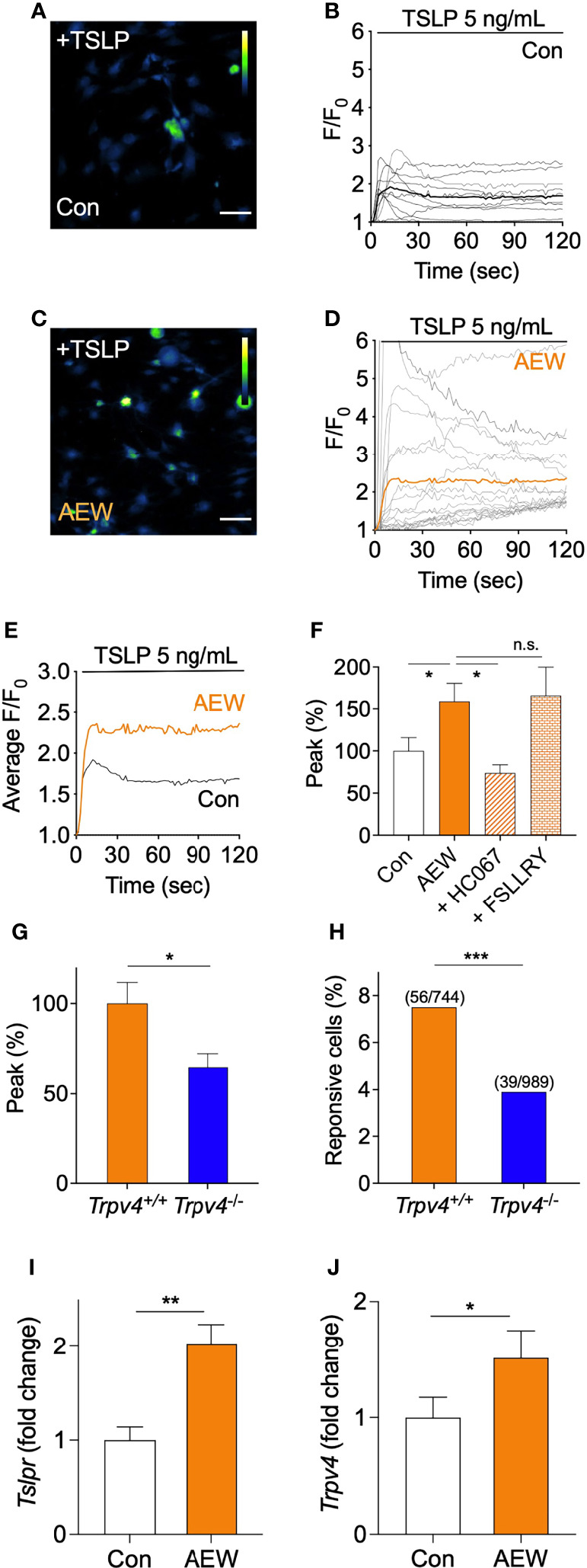
The sensory neurons of AEW-treated mice showed increased responses to TSLP, in a TRPV4-dependent manner. **(A)** Representative calcium imaging fluorescent responses following the application of 5 ng/mL TSLP to the primary culture of murine DRG neurons of the control mice. The scale bar indicates 50 µm. **(B)** A time-course graph of TSLP-induced calcium-specific fluorescence in the DRG neurons from the control mice following treatment with TSLP (n = 10). F denotes the fluorescence intensity of the region of interest, and F_0_ indicates the initial fluorescence intensity. **(C)** Representative calcium imaging fluorescent responses following the application of TSLP in the primary culture of DRG neurons of AEW-treated mice. **(D)** A time-course graph of TSLP-induced calcium-specific fluorescence, indicating the responses of the DRG neurons of AEW-treated mice following treatment with TSLP (n = 19). **(E)** Averaged time-course profiles of TSLP-induced responses of the DRG neurons of the control (n = 10) and AEW-treated (n = 19) mice. **(F)** Pretreatment of the DRG neurons of AEW-treated mice with the TRPV4 antagonist, HC06747, significantly inhibited the TSLP-induced responses (n = 21), while treatment with the PAR2 antagonist, FSLLRY, produced no changes (n = 5). **(G)** The TSLP-induced responses of the DRG neurons of *Trpv4* ^-/-^ mice (n = 39) revealed that the peak responses were significantly reduced in comparison to those of the control (n = 56). **(H)** The percentage of TSLP-responsive neurons in *Trpv4* ^-/-^ mice (3.9%, 39 out of total 989 neurons) was significantly lower than that of *Trpv4* ^+/+^ mice (7.5%, 56 out of total 744 neurons) (Fisher’s exact test). Transcriptional levels of both *Tslpr* **(I)** and *Trpv4* **(J)** were increased in DRG from AEW-treated mice (n = 3 per group). *p < 0.05, **p < 0.01, ***p < 0.001, n.s. not significant.

TSLP is known to excite the innervated sensory neurons by binding to its receptor, TSLPR, which triggers an increase in the intracellular levels of calcium required for itch transmission (4). Thus, the dorsal root ganglia (DRG) neurons from the control and AEW-treated mice were primarily cultured, and the responses to TSLP were measured using calcium imaging techniques. The results demonstrated that the DRG neurons of AEW-treated mice exhibited stronger responses to TSLP treatment (**Figures 5C, D**) than those of the control mice (**Figures 5A, B**). Additionally, the average fluorescence intensities of the DRG neurons increased significantly in the AEW-treated mice compared to those of the control (**Figure 5E**). The pretreatment of DRG neurons with the TRPV4 antagonist, HC067047, significantly suppressed the peak response to TSLP in AEW-treated mice (**Figure 5F**), suggesting that TRPV4 has a specific role in the sensory neurons of AEW-treated mice. Pretreatment with HC030031, a TRPA1 antagonist, also significantly inhibited the peak responses to TSLP (**Figure S6A**). For comparison, the pretreatment of DRG neurons of AEW-treated mice with either FSLLRY (a PAR2 antagonist, **Figure 5F**) or capsazepine (a TRPV1 antagonist, **Figure S6B**) did not alter the peak responses induced by TSLP. Similar experiments were performed using the DRG neurons of AEW-treated *Trpv4^-/-^* mice. The results demonstrated that both the peak responses (**Figure 5G**) and responsive cell percentage (**Figure 5H**) following TSLP treatment decreased significantly in the DRG neurons of *Trpv4^-/-^* mice. Importantly, it was observed that the transcriptional levels of both *Tslpr* (**Figure 5I**) and *Trpv4* (**Figure 5J**) were increased in the DRG neurons from AEW-treated mice. Therefore, these data imply that the AEW treatment may sensitize DRG neurons to TSLP in a TRPV4-dependent manner.

### The TRPV4-Mediated Production of TSLP Increases Mast Cell Proliferation and Degranulation

**Figure 6:**
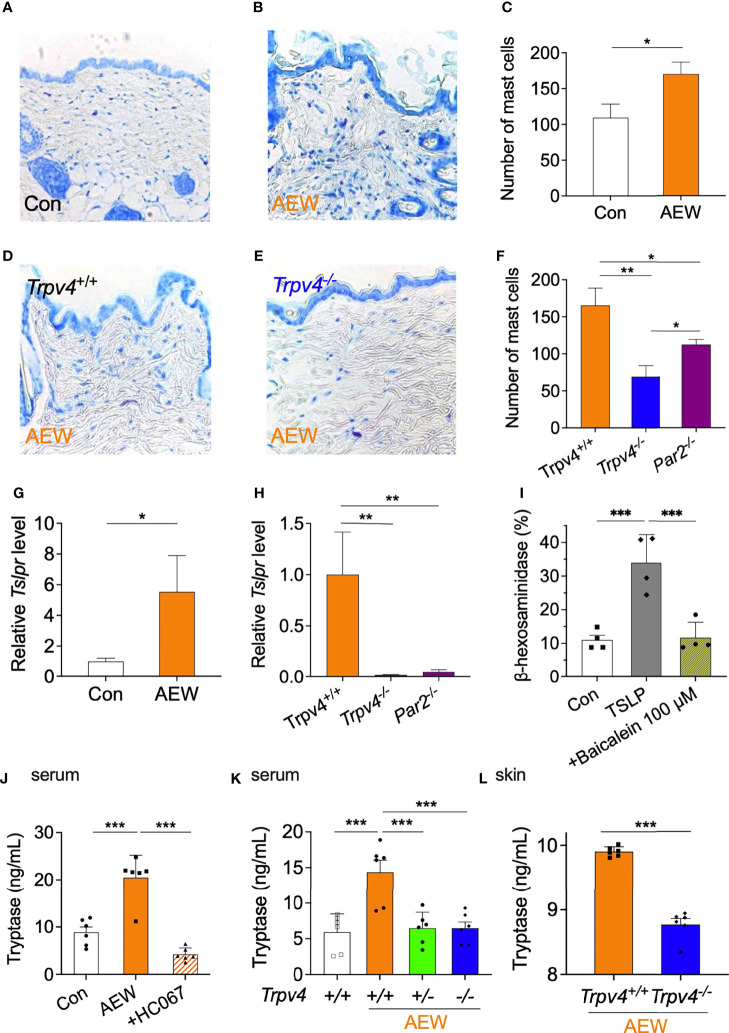
The TRPV4-mediated production of TSLP affects the increased proliferation and degranulation of mast cells. Representative toluidine blue-stained images of the skin tissues of the control **(A)** and AEW-treated mice **(B)**. **(C)** The number of mast cells significantly increased in the skin tissues of AEW-treated mice (n = 3 per group). Representative toluidine blue-stained images of the skin tissues of AEW-treated *Trpv4* ^+/+^ **(D)** and *Trpv4^-/-^* mice **(E)** are depicted. **(F)** Comparison of the number of mast cells in *Trpv4* ^+/+^, *Trpv4^-/-^*, and *Par2^-/-^* mice (n = 3 per group). The number of mast cells was lowest in the *Trpv4^-/-^* mice following treatment with AEW. **(G)** The transcriptional levels of *Tslpr* were significantly increased in the mast cells of AEW-treated mice (n = 3 per group). **(H)** The transcriptional levels of *Tslpr* were not elevated in the mast cells of *Trpv4^-/-^* and *Par2^-/-^* mice (n = 3 per group). **(I)** Treatment with TSLP induced mast cell degranulation, as revealed by measuring the release of β-hexosaminidase. Mast cell degranulation was inhibited by baicalein, a TSLPR inhibitor (n = 4 per group). **(J)** The serum levels of tryptase were elevated in the AEW mice, and were significantly reduced when the TRPV4 antagonist, HC067047, was administered during AEW treatment (n = 6 per group). **(K)** The serum levels of tryptase were significantly reduced in both the AEW-treated *Trpv4^+/-^* mice and *Trpv4^-/-^* mice (n = 6 per group). **(L)** The tryptase levels in the skin tissues of AEW-treated *Trpv4^-/-^* mice were lower than those of the AEW-treated *Trpv4* ^+/+^ mice (n = 6 per group). *p < 0.05, **p < 0.01, ***p < 0.001.

As TSLP can induce mast cell development and aggravate allergic reactions (15), putative changes in the mast cells of AEW-treated mice were also investigated. We observed that the number of mast cells in the skin of AEW-treated mice significantly increased in comparison to that of the control mice (**Figures 6A–C**), suggesting that TSLP has a proliferative role in mast cell development. In contrast, the treatment of *Trpv4^-/-^* mice with AEW did not increase the number of mast cells in the skin compared to that of *Trpv4^+/+^* mice (**Figures 6D–F**). The number of mast cells also decreased in AEW-treated *Par2* KO (*Par2^-/-^*) mice (**Figure 6F**). However, the reduction in the number of mast cells was greater in *Trpv4^-/-^* mice compared to that in *Par2^-/-^* mice (**Figure 6F**), implying that TRPV4 has a stronger effect on the proliferation of mast cells than PAR2 following AEW treatment. There were no species-specific differences between the ICR and C57BL/6 mice following treatment with AEW, in terms of the number of mast cells (**Figure S6**). Comparison of the transcriptional levels of *Tslpr* in the mast cells of AEW-treated and control mice revealed that the levels of *Tslpr* significantly increased in the mast cells of AEW-treated mice, compared to those of the control (**Figure 6G**). Moreover, the transcriptional levels of *Tslpr* were hardly detected in the mast cells of *Trpv4^-/-^* mice treated with AEW (**Figure 6H**), suggesting that treatment with AEW induced an overexpression of TSLPR in the mast cells, probably *via* TRPV4. The transcriptional levels of *Tslpr* also significantly decreased in *Par2^-/-^* mice treated with AEW (**Figure 6H**), implying that *Par2* plays a role in the proliferation of mast cells as well. As shown in **Figure 6I**, a mast cell degranulation assay confirmed that TSLP could induce mast cell degranulation, which was inhibited by the TSLPR inhibitor, baicalein (16). This implied that the TSLP-induced degranulation of mast cells is mediated by TSLPR in mast cells.

Tryptase is a protease and PAR2 agonist, that is released during mast cell degranulation. We therefore measured the levels of tryptase in the AEW-treated mice. As depicted in **Figure 6J**, the serum levels of tryptase significantly increased in the AEW-treated mice compared to those of the control. However, the serum levels of tryptase reduced following the administration of the TRPV4-specific antagonist, HC067047, to AEW-treated mice (**Figure 6J**). Meanwhile, the serum levels of tryptase did not increase in *Trpv4^+/-^* and *Trpv4^-/-^* mice treated with AEW (**Figure 6K**). Indeed, the tryptase levels in the skin of AEW-treated *Trpv4^-/-^* mice also reduced in comparison to those of the AEW-treated *Trpv4^+/+^* mice (**Figure 6L**). Taken together, these data suggest that the TRPV4-induced production of TSLP from keratinocytes induced mast cell degranulation, which might have released tryptase following treatment with AEW.

### Inhibition of TSLPR Selectively Reduced Scratching Behaviors in AEW-Treated Mice

**Figure 7:**
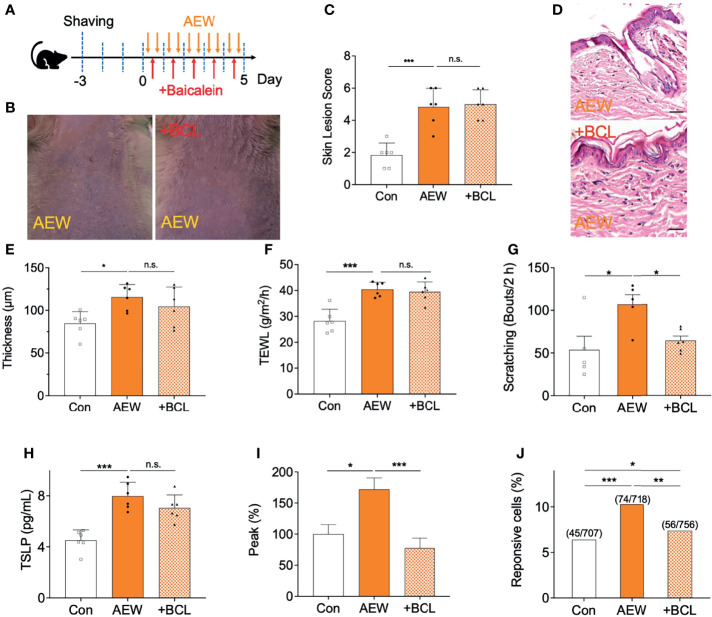
Administration of a TSLPR antagonist reduced scratching behaviors in AEW-treated mice. **(A)** A schematic illustration depicting the administration of baicalein (BCL) during treatment with AEW. **(B)** Pretreatment with BCL did not alleviate the overall skin conditions following AEW treatment. **(C)** The skin lesion scores are not reduced in the BCL-treated AEW mice on day 5 (n = 6). **(D)** Representative H&E-stained images of the skin lesions of AEW-treated mice with or without BCL administration. **(E)** The thickness of the epidermis did not change in the skin lesions of the BCL-treated AEW mice (n = 6) **(F)** The values of TEWL were not altered in the BCL-treated AEW mice on day 5 (n = 6). **(G)** The spontaneous scratching bouts were significantly reduced in the BCL-treated AEW mice (n = 6), compared to those of the AEW mice (n = 6). **(H)** The serum levels of TSLP did not decrease in the BCL-treated AEW mice (n = 6), compared to those of the AEW-treated mice (n = 6). **(I)** The TSLP-induced responses of the DRG neurons from BCL-treated AEW mice (n = 56) revealed that the peak responses were significantly reduced in comparison to those of the AEW mice (n = 76). **(J)** The percentage of TSLP-responsive neurons in BCL-treated AEW mice (7.4%, 56 out of total 756 neurons) were significantly lower than those of the AEW mice (10.3%, 74 out of total 718 neurons) (Fisher’s exact test). *p < 0.05, **p < 0.01, ***p < 0.001, n.s. not significant.

Finally, it was confirmed whether scratching behaviors in AEW mice are related to the activation of TSLPR in sensory neurons. For this reason, a TSLPR inhibitor baicalein (BCL, 50 mg/kg) was administered daily during AEW treatment (**Figure 7A**), and the changes were compared with control groups. As shown in **Figures 7B, C**, however, BCL treatment did not improve the skin lesions induced by AEW treatment. Similarly, the effect of BCL administration in AEW-treated mice had no impact on the epidermal thickness (**Figures 7D, E**). The TEWL values also revealed that BCL treatment has no effect on the dry skin conditions (**Figure 7F**). However, BCL treatment selectively reduced spontaneous scratching behaviors in AEW-treated mice (**Figure 7G**), suggesting that AEW-induced pruritus related to the activation of TSLPR. On the other hand, BCL-administered AEW mice showed similar serum TSLP levels compared to AEW-treated mice (**Figure 7H**), implying that the production of TSLP is not affected by inhibition of TSLPR. When calcium imaging experiments with DRG neurons from BCL-administered AEW-treated mice were performed, it was found that TSLP-induced peak responses (**Figure 7I**) as well as the percentage of responsive cells (**Figure 7J**) were significantly decreased, suggesting that TSLPR is selectively related to the transmission of TSLP-induced pruritus signal in the sensory neuron. Overall, these data imply that TSLPR in the sensory neuron is selectively responsible for the AEW-induced pruritus.

## Discussion

Dry skin is a common condition that occurs in several dermatological diseases, including xerosis (often referred to as abnormally dry skin), atopic dermatitis, and psoriasis. Dry skin is also an accompanying symptom of systemic diseases, including chronic kidney disease, chronic liver disease, and diabetes mellitus (1). From a molecular perspective, dry skin is thought to occur when the functions of genes related to natural moisturizing factors (NMFs) are altered. For instance, the mutation or deficiency of the filaggrin gene, *FLG*, which preserves skin integrity and the production of NMF, is tightly linked to the development of dry skin (17). Similarly, the aquaporin-3 gene, *AQP3*, is involved in transepithelial water transport, and its overexpression often induces the onset of dry skin (18). On the other hand, environmental factors such as frequent washing or showering and low humidity are also related to the development of dry skin (19), which explains the higher prevalence of dry skin in winter.

The problem of dry skin is that it not only damages the barrier function of the skin, but also provokes pruritus that induces scratching behavior, which may lead to desquamation or inflammation of the stratum corneum, thus further aggravating the damage to the barrier function of the skin. However, the molecular and cellular mechanisms by which dry skin induce pruritus remains to be further investigated. It is expected that either the sensitivity of the itch-sensitive neurons is elevated or the production of pruritogen(s) increases under dry skin conditions. Studies investigating the alterations in neuronal sensitivity have consistently reported that the intraepithelial nerve density increases in dry skin-related diseases (20–22). Detailed analyses have revealed that the balance between an increase in nerve elongation factors, such as NGF, and a decrease in the nerve repulsion factors, such as Sema3A, is the key to maintaining cutaneous nerve density. In fact, dry skin conditions appear to occur due to a net increase in nerve elongation signals, which increase the density of intraepithelial itch-sensitive neurons in the afflicted skin areas. However, it is also possible that the production of pruritogen(s) also increases under dry skin conditions. Although histamine is the most widely studied endogenous pruritogen, there is a consensus that dry skin-induced pruritus is not mediated by histamine, as antihistamines are not completely effective in various dry skin-related diseases (23). We therefore speculated that there might be certain non-histamine pruritogen(s) that are overproduced in dry skin conditions.

Based on the results of previous studies, we presumed that TSLP may be involved in dry skin-induced pruritus. Indeed, the present study clearly demonstrated that AEW-induced dry skin conditions evoked the elevated production of TSLP in mouse skin (**Figure 1**). The fact that TSLP is not associated with the histamine-dependent itch pathway is consistent with a previous report which demonstrated that antihistamines are ineffective against dry skin-induced pruritus (23). On the other hand, the transcriptional levels of IL-33 were also increased in the AEW-treated mice (**Figure S2**). Previous studies have reported that the production of IL-33 increases in dry skin conditions resulting from allergic contact dermatitis, atopic dermatitis, and animal models of skin trauma caused by tape-stripping (24–26). Although we cannot rule out the possibility that IL-33 is involved in dry skin pruritus, it was speculated that the role of IL-33 in AEW-treated mice does not seem strongly related to TRPV4 because transcriptional levels of IL-33 also increased in the HC067047-administered AEW mice (**Figure S2D**) as well as *Trpv4* ^-/-^ AEW mice (**Figure S2E**).

Although TRPV1 and TRPA1 are the major TRP ion channels that are directly involved in various types of pruritus, increasing evidence suggests that TRPV4 also plays an essential role in pruritus (27). For instance, a recent study highlighted the importance of TRPV4 in cinnamaldehyde-evoked scratching behaviors in mice (28). Additionally, the occurrence of pruritus in psoriasis appears to be associated with TRPV4 (29). As TRPV4 is mostly expressed in the peripheral sensory neurons (30) and keratinocytes (6, 31), it is not surprising that TRPV4 plays a significant role in the AEW-induced dry skin pruritus found in the present study.

TSLP binds to the TSLP receptor complex on the cell surface, which comprises two subunits, namely, TSLPR (encoded by *CRLF2*) and IL-7Rα (32, 33). As TSLP is considered to be a distant paralog of IL-7, it is not surprising that IL-7Rα is a component of the TSLP receptor complex. However, it is necessary to mention that IL-7Rα alone does not evoke the signals induced by TSLP. In other words, the TSLPR subunit functions as a unique molecular entity in evoking TSLP-specific signaling. TSLPR is expressed not only in the sensory neurons, but also in numerous immune cells, including immature dendritic cells, basophils, and mast cells (34). The TSLP-TSLPR signaling pathway appears to be selective, as the TSLP-induced responses remained unaltered following pretreatment with the PAR2 antagonist, FSLLRY (**Figure 5F**).

**Figure 8:**
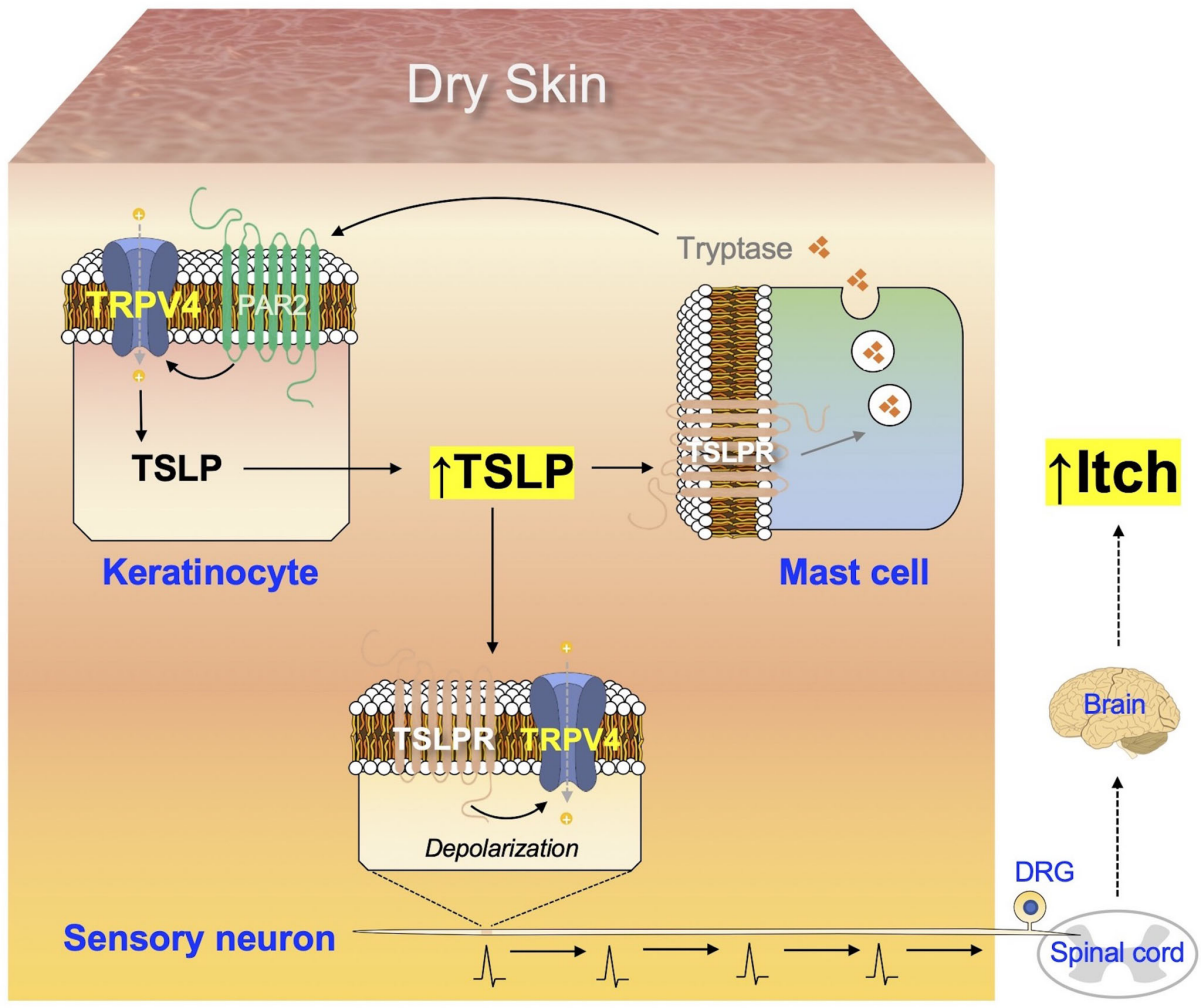
Summary of the mechanism underlying dry skin-induced pruritus. The production of TSLP in keratinocytes increases under dry skin conditions, in a TRPV4-dependent manner. Additionally, TSLP acts on TSLPR in mast cells to promote the release of tryptase, which in turn stimulates the keratinocytes *via* PAR2 and TRPV4 to produce more TSLP, thus generating a positive feedback loop of increased TSLP production. TSLP can also act on TSLPR in sensory neurons to transmit electrical signals towards the spinal cord, which are conveyed to the brain and finally perceived as itch.

In the present study, activation of the TSLP-TSLPR signaling pathway resulted in the generation of signals in DRG neurons by TRPV4-dependent manner (**Figure 5**). Moreover, we observed that the alterations induced by AEW in mast cells were TSLPR-dependent (**Figure 6**), suggesting that the TSLP-TSLPR signaling pathway also plays a role in mast cells. More importantly, it was also found that the TSLP-TSLPR signaling pathway is specifically involved in the spontaneous scratching behavior in AEW mice (**Figure 7**). Although the present study may have limitations such as small sample sizes and non-optimal experimental designs, we present a mechanism underlying the onset of pruritus in dry skin conditions, illustrated in **Figure 8**. Dry skin induces the elevated production of TSLP in keratinocytes in a TRPV4-dependent manner. TSLP triggers mast cell degranulation *via* TSLPR to promote the release of tryptase. The released tryptase stimulates PAR2, and subsequently TRPV4 in keratinocytes to produce TSLP. While this generates a positive feedback loop of enhanced TSLP production, TSLP can also activate the innervated peripheral sensory neurons through TSLPR and TRPV4, thereby producing electrical signals for the onset of pruritus. The signal is transmitted into the spinal cord and ascends to the brain, to be finally perceived as pruritus. The present study suggests the importance of TSLP and TRPV4 in dry skin conditions and identified potential molecular targets for dry skin-induced pruritus.

## Data Availability Statement

The raw data supporting the conclusions of this article will be made available by the authors, without undue reservation.

## Ethics Statement

The animal study was reviewed and approved by The Institutional Animal Care and Use Committee of Gachon University (GIACUC-R2020002).

## Author Contributions

W-JL and W-SS conceptualized the study. W-JL investigated the study. W-JL and W-SS wrote the manuscript. W-SS supervised the study. All authors contributed to the article and approved the submitted version.

## Conflict of Interest

The authors declare that the research was conducted in the absence of any commercial or financial relationships that could be construed as a potential conflict of interest.

## Publisher’s Note

All claims expressed in this article are solely those of the authors and do not necessarily represent those of their affiliated organizations, or those of the publisher, the editors and the reviewers. Any product that may be evaluated in this article, or claim that may be made by its manufacturer, is not guaranteed or endorsed by the publisher.

## References

[1] Moniaga CS Tominaga M Takamori K. Mechanisms and Management of Itch in Dry Skin. Acta Derm Venereol (2020) 100(2):adv00024. doi: 10.2340/00015555-3344 31940044 PMC9129002

[2] Miyamoto T Nojima H Shinkado T Nakahashi T Kuraishi Y. Itch-Associated Response Induced by Experimental Dry Skin in Mice. Jpn J Pharmacol (2002) 88(3):285–92. doi: 10.1254/jjp.88.285 11949883

[3] Ziegler SF. Thymic Stromal Lymphopoietin and Allergic Disease. J Allergy Clin Immunol (2012) 130(4):845–52. doi: 10.1016/j.jaci.2012.07.010 PMC3462264 22939755

[4] Wilson SR The L Batia LM Beattie K Katibah GE McClain SP. The Epithelial Cell-Derived Atopic Dermatitis Cytokine TSLP Activates Neurons to Induce Itch. Cell (2013) 155(2):285–95. doi: 10.1016/j.cell.2013.08.057 PMC4041105 24094650

[5] White JP Cibelli M Urban L Nilius B McGeown JG Nagy I. TRPV4: Molecular Conductor of a Diverse Orchestra. Physiol Rev (2016) 96(3):911–73. doi: 10.1152/physrev.00016.2015 27252279

[6] Luo J Feng J Yu G Yang P Mack MR Du J. Transient Receptor Potential Vanilloid 4-Expressing Macrophages and Keratinocytes Contribute Differentially to Allergic and Nonallergic Chronic Itch. J Allergy Clin Immunol (2018) 141(2):608–19.e607. doi: 10.1016/j.jaci.2017.05.051 28807414 PMC5799031

[7] Mizuno A Matsumoto N Imai M Suzuki M. Impaired Osmotic Sensation in Mice Lacking TRPV4. Am J Physiol Cell Physiol (2003) 285(1):C96–101. doi: 10.1152/ajpcell.00559.2002 12777254

[8] Suzuki M Mizuno A Kodaira K Imai M. Impaired Pressure Sensation in Mice Lacking TRPV4. J Biol Chem (2003) 278(25):22664–8. doi: 10.1074/jbc.M302561200 12692122

[9] Sanjel B Maeng HJ Shim WS. BAM8-22 and its Receptor MRGPRX1 may Attribute to Cholestatic Pruritus. Sci Rep (2019) 9(1):10888. doi: 10.1038/s41598-019-47267-5 31350433 PMC6659683

[10] Li F Adase CA Zhang LJ. Isolation and Culture of Primary Mouse Keratinocytes From Neonatal and Adult Mouse Skin. J Vis Exp (2017) 125:56027. doi: 10.3791/56027 PMC5612483 28745643

[11] Tsvilovskyy V Solis-Lopez A Ohlenschlager K Freichel M. Isolation of Peritoneum-Derived Mast Cells and Their Functional Characterization With Ca2+-Imaging and Degranulation Assays. J Vis Exp (2018) 137:57222. doi: 10.3791/57222 PMC6102043 30035759

[12] Wilson SR Nelson AM Batia L Morita T Estandian D Owens DM. The Ion Channel TRPA1 is Required for Chronic Itch. J Neurosci (2013) 33(22):9283–94. doi: 10.1523/JNEUROSCI.5318-12.2013 PMC3752436 23719797

[13] Kouzaki H O'Grady SM Lawrence CB Kita H. Proteases Induce Production of Thymic Stromal Lymphopoietin by Airway Epithelial Cells Through Protease-Activated Receptor-2. J Immunol (2009) 183(2):1427–34. doi: 10.4049/jimmunol.0900904 PMC2706924 19561109

[14] Um JY Kang SY Kim HJ Chung BY Park CW Kim HO. Transient Receptor Potential Vanilloid-3 (TRPV3) Channel Induces Dermal Fibrosis via the TRPV3/TSLP/Smad2/3 Pathways in Dermal Fibroblasts. J Dermatol Sci (2020) 97(2):117–24. doi: 10.1016/j.jdermsci.2019.12.011 31959383

[15] Han NR Oh HA Nam SY Moon PD Kim DW Kim HM. TSLP Induces Mast Cell Development and Aggravates Allergic Reactions Through the Activation of MDM2 and STAT6. J Invest Dermatol (2014) 134(10):2521–30. doi: 10.1038/jid.2014.198 24751726

[16] Park BB Choi JW Park D Choi D Paek J Kim HJ. Structure-Activity Relationships of Baicalein and its Analogs as Novel TSLP Inhibitors. Sci Rep (2019) 9(1):8762. doi: 10.1038/s41598-019-44853-5 31217492 PMC6584507

[17] Sandilands A Sutherland C Irvine AD McLean WH. Filaggrin in the Frontline: Role in Skin Barrier Function and Disease. J Cell Sci (2009) 122(Pt 9):1285–94. doi: 10.1242/jcs.033969 PMC2721001 19386895

[18] Hara-Chikuma M Verkman AS. Roles of Aquaporin-3 in the Epidermis. J Invest Dermatol (2008) 128(9):2145–51. doi: 10.1038/jid.2008.70 18548108

[19] Proksch E Berardesca E Misery L Engblom J Bouwstra J. Dry Skin Management: Practical Approach in Light of Latest Research on Skin Structure and Function. J Dermatolog Treat (2020) 31(7):716–22. doi: 10.1080/09546634.2019.1607024 30998081

[20] Tominaga M Ozawa S Tengara S Ogawa H Takamori K. Intraepidermal Nerve Fibers Increase in Dry Skin of Acetone-Treated Mice. J Dermatol Sci (2007) 48(2):103–11. doi: 10.1016/j.jdermsci.2007.06.003 17643268

[21] Kamo A Tominaga M Negi O Tengara S Ogawa H Takamori K. Topical Application of Emollients Prevents Dry Skin-Inducible Intraepidermal Nerve Growth in Acetone-Treated Mice. J Dermatol Sci (2011) 62(1):64–6. doi: 10.1016/j.jdermsci.2011.01.008 21316926

[22] Tominaga M Takamori K. Itch and Nerve Fibers With Special Reference to Atopic Dermatitis: Therapeutic Implications. J Dermatol (2014) 41(3):205–12. doi: 10.1111/1346-8138.12317 24628070

[23] Kamo A Tominaga M Kamata Y Takamori K. Mechanisms and Treatments of Dry Skin-Induced Itch. J Clin Cosmet Dermatol (2017) 1(3). doi: 10.16966/2576-2826.114

[24] Dickel H Gambichler T Kamphowe J Altmeyer P Skrygan M. Standardized Tape Stripping Prior to Patch Testing Induces Upregulation of Hsp90, Hsp70, IL-33, TNF-Alpha and IL-8/CXCL8 mRNA: New Insights Into the Involvement of 'Alarmins'. Contact Dermatitis (2010) 63(4):215–22. doi: 10.1111/j.1600-0536.2010.01769.x 20731692

[25] Liu B Tai Y Achanta S Kaelberer MM Caceres AI Shao X. IL-33/ST2 Signaling Excites Sensory Neurons and Mediates Itch Response in a Mouse Model of Poison Ivy Contact Allergy. Proc Natl Acad Sci USA (2016) 113(47):E7572–9. doi: 10.1073/pnas.1606608113 PMC5127381 27821781

[26] Pietka W Khnykin D Bertelsen V Lossius AH Stav-Noraas TE Hol Fosse J. Hypo-Osmotic Stress Drives IL-33 Production in Human Keratinocytes-An Epidermal Homeostatic Response. J Invest Dermatol (2019) 139(1):81–90. doi: 10.1016/j.jid.2018.07.023 30120934

[27] Zhang Q Henry G Chen Y. Emerging Role of Transient Receptor Potential Vanilloid 4 (TRPV4) Ion Channel in Acute and Chronic Itch. Int J Mol Sci (2021) 22(14):7591. doi: 10.3390/ijms22147591 34299208 PMC8307539

[28] Domocos D Follansbee T Nguyen A Nguyen T Carstens MI Carstens E. Cinnamaldehyde Elicits Itch Behavior via TRPV1 and TRPV4 But Not TRPA1. Itch (2020) 5(3):e36. doi: 10.1097/itx.0000000000000036 34458578 PMC8388579

[29] Yan J Ye F Ju Y Wang D Chen J Zhang X. Cimifugin Relieves Pruritus in Psoriasis by Inhibiting TRPV4. Cell Calcium (2021) 97:102429. doi: 10.1016/j.ceca.2021.102429 34087722

[30] Kim S Barry DM Liu XY Yin S Munanairi A Meng QT. Facilitation of TRPV4 by TRPV1 is Required for Itch Transmission in Some Sensory Neuron Populations. Sci Signal (2016) 9(437):ra71. doi: 10.1126/scisignal.aaf1047 27436359 PMC5310287

[31] Chen Y Fang Q Wang Z Zhang JY MacLeod AS Hall RP. Transient Receptor Potential Vanilloid 4 Ion Channel Functions as a Pruriceptor in Epidermal Keratinocytes to Evoke Histaminergic Itch. J Biol Chem (2016) 291(19):10252–62. doi: 10.1074/jbc.M116.716464 PMC4858974 26961876

[32] Pandey A Ozaki K Baumann H Levin SD Puel A Farr AG. Cloning of a Receptor Subunit Required for Signaling by Thymic Stromal Lymphopoietin. Nat Immunol (2000) 1(1):59–64. doi: 10.1038/76923 10881176

[33] Park LS Martin U Garka K Gliniak B Di Santo JP Muller W. Cloning of the Murine Thymic Stromal Lymphopoietin (TSLP) Receptor: Formation of a Functional Heteromeric Complex Requires Interleukin 7 Receptor. J Exp Med (2000) 192(5):659–70. doi: 10.1084/jem.192.5.659 PMC2193276 10974032

[34] He R Geha RS. Thymic Stromal Lymphopoietin. Ann N Y Acad Sci (2010) 1183:13–24. doi: 10.1111/j.1749-6632.2009.05128.x 20146705 PMC2895428

