# Supplementary material for: Cutaneous Neuroimmune Interactions of TSLP and TRPV4 Play Pivotal Roles in Dry Skin-Induced Pruritus
Source: Front Immunol. 2021 Dec 2;12:772941. doi: 10.3389/fimmu.2021.772941 (PMC8674573; doi:10.3389/fimmu.2021.772941)
Supplement: Supplementary file 1 [file DataSheet_1.pdf]

Supplementary Material

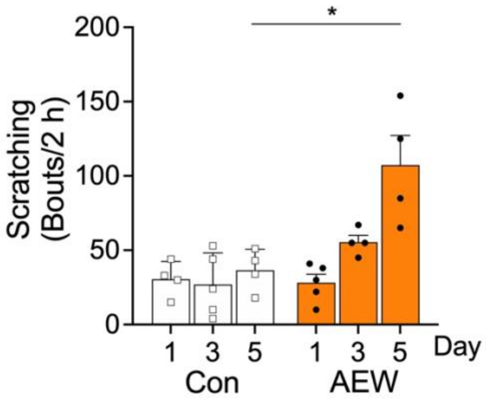

Figure S1. Spontaneous scratching behaviors of AEW-treated mice on days 1, 3, and 5. * $p < 0.05$

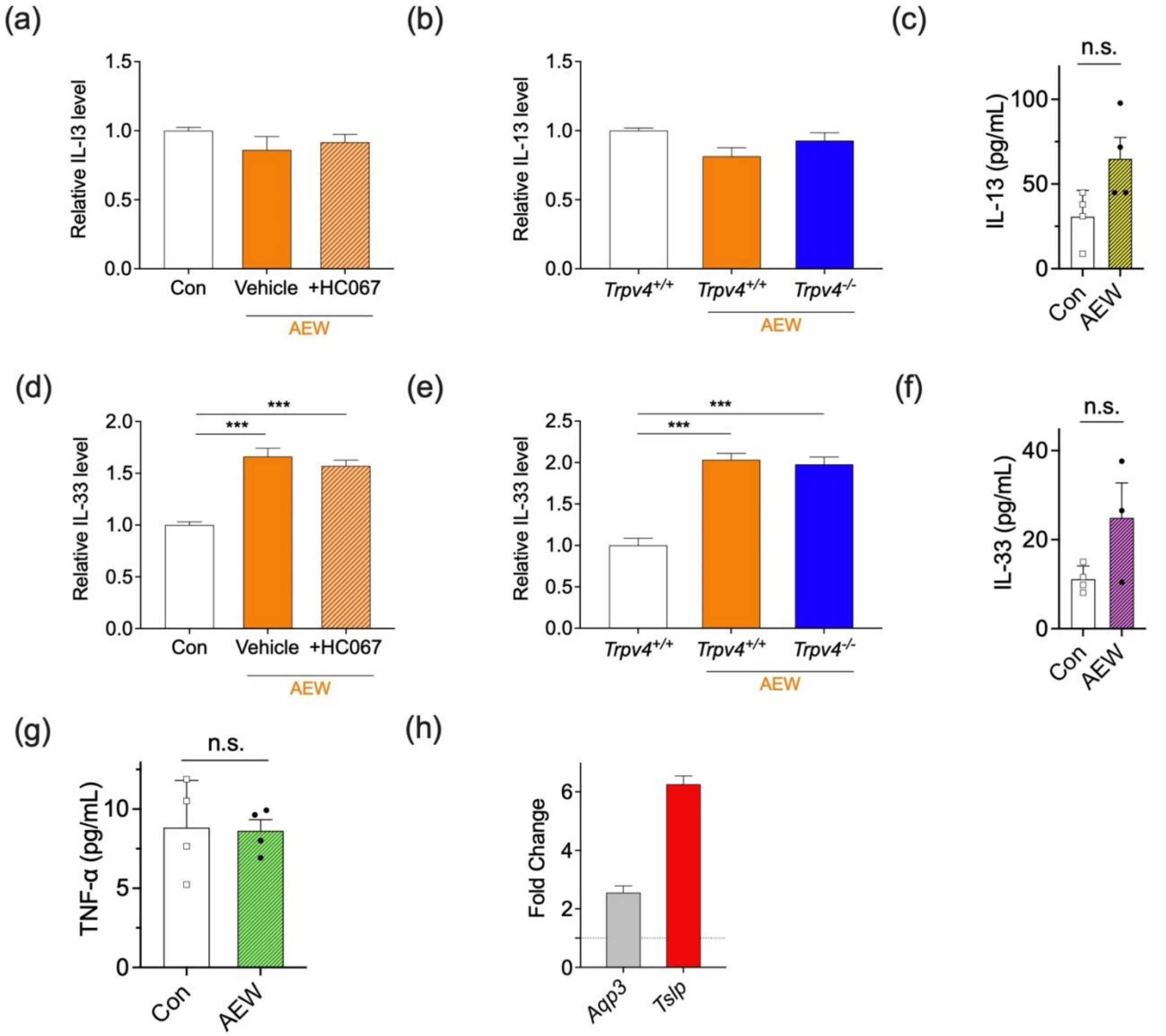

Figure S2. (a) Transcriptional levels of IL-13 in the skin of HC067047-administered AEW ICR mice administered by HC067047 ("+HC067") *vs.* control groups (n = 3) (b) Transcriptional levels of IL-13 in the skin of different genotypes (n = 3) (c) Serum levels of IL-13 between AEW-treated and control mice (n = 3) (d) Transcriptional levels of IL-33 in the skin of AEW ICR mice ("+HC067") *vs.* control groups (n = 3) (e) Transcriptional levels of IL-33 in the skin of different genotypes (n = 3) (f) Serum levels of IL-33 between AEW-treated and control mice (n = 3) (g) serum levels of TNF-α (n = 4) between AEW-treated and control mice. (h) The transcriptional changes in *Aqp3* and *Tslp* in murine keratinocytes on day 5 after treatment with AEW. *** $p < 0.001$, *n.s.* not significant

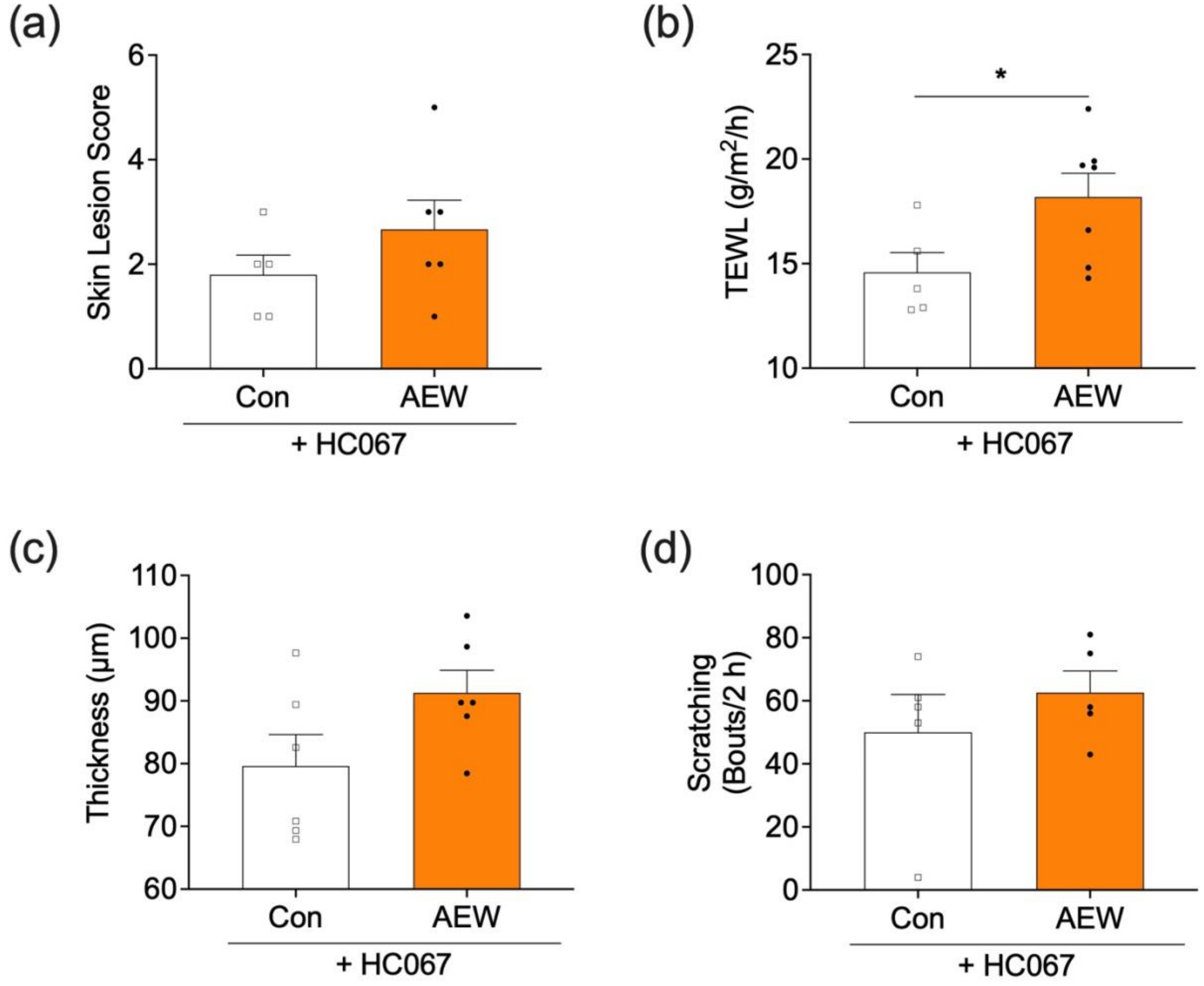

Figure S3. The effect of HC067047 administration ("+HC067") on the skin lesion scores (a), TEWL (b), skin thickness (c), and spontaneous scratching bouts (d) between control and AEW-treated mice. * $p < 0.05$

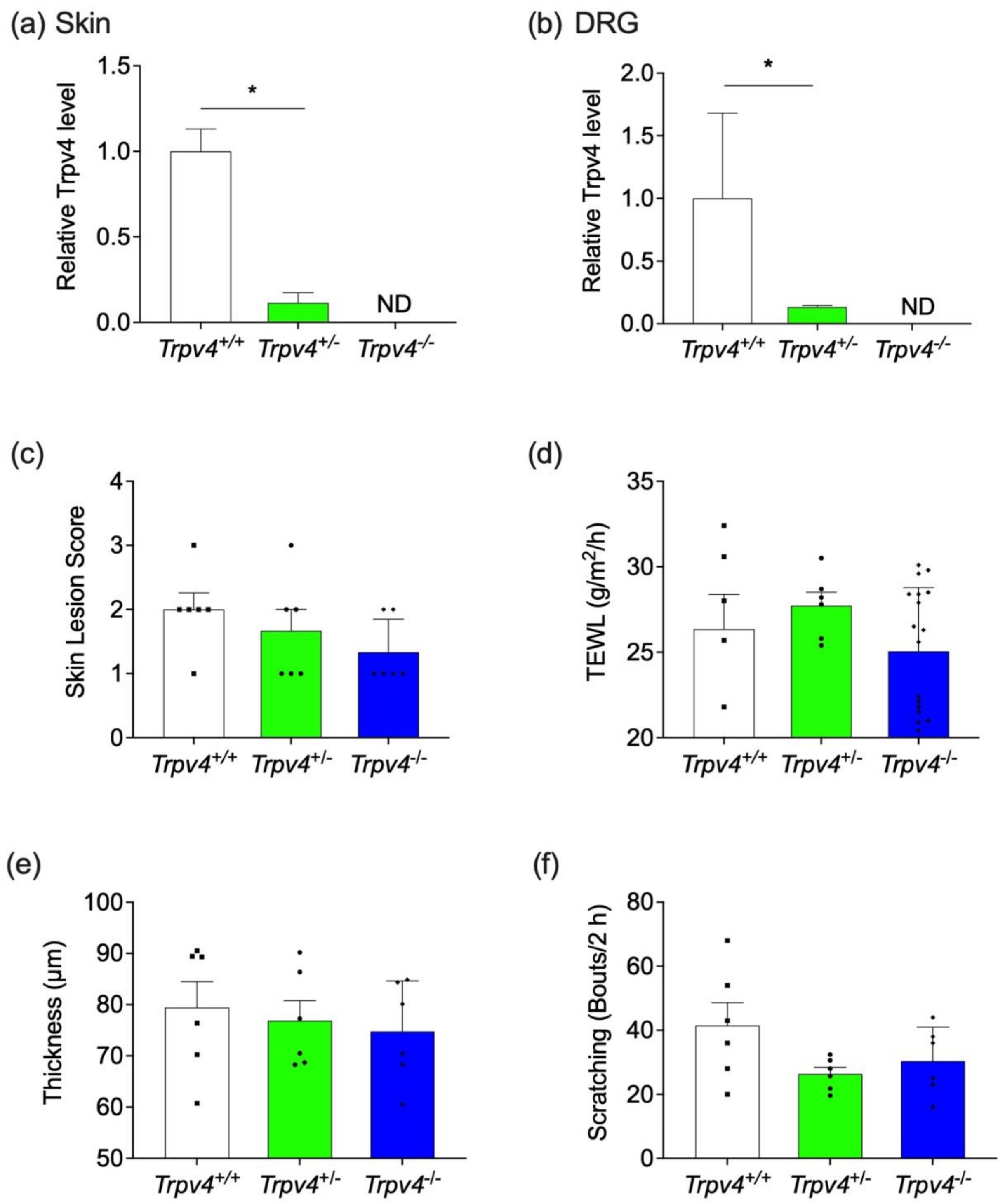

Figure S4. Comparisons of *Trpv4* levels in the skin (a) and the DRG among different *Trpv4* genotype mice. Differences in skin lesion scores (c), TEWL (d), skin thickness (e) and spontaneous scratching bouts (f) between different *Trpv4* genotype mice. * $p < 0.05$, ND: not detected

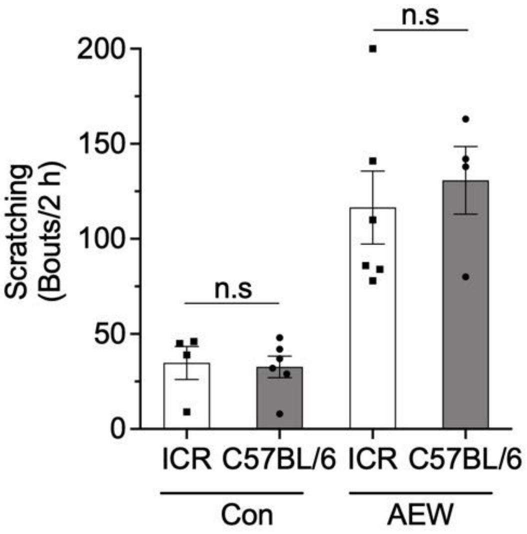

Figure S5. Effect of AEW treatment on spontaneous scratching behaviors of different strains of mice (ICR vs. C57BL/6). There were no significant differences between the spontaneous scratching behaviors of the two strains (n = 4~6 per group).

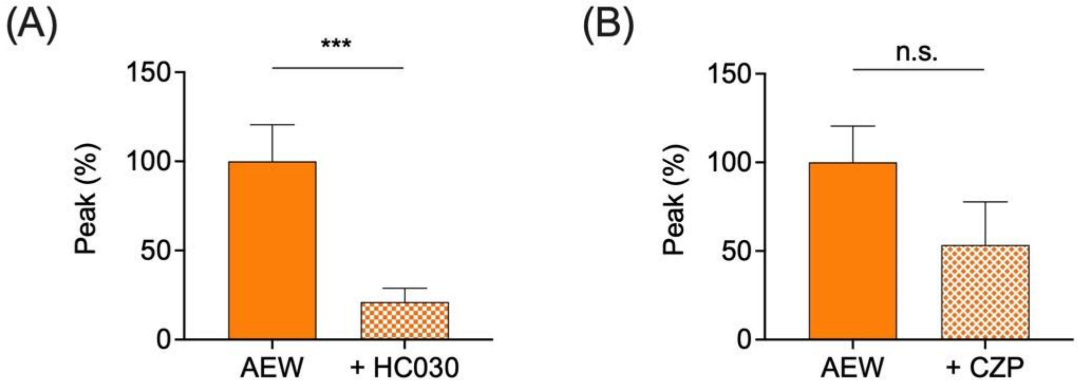

Figure S6. (A) Pretreatment with 100 µM HC030031, a specific TRPA1 antagonist, significantly inhibited the peak responses induced by 5 ng/mL TSLP in the DRG neurons of AEW-treated mice (n = 65), in comparison to those of the control (n = 27). (B) Pretreatment with 10 µM capsazepine, a TRPV1 antagonist, produced no differences in the peak responses induced by TSLP in the DRG neurons of AEW-treated mice (n = 15), in comparison to those of the control (n = 27). *** $p < 0.001$, n.s. not significant.

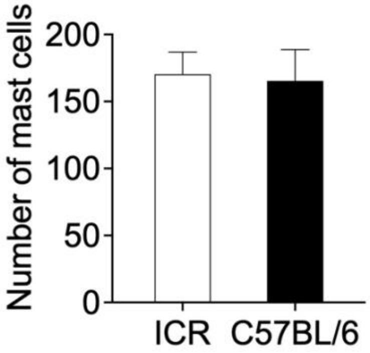

Figure S7. The average number of mast cells in ICR (170.3 ± 9.528, n = 3) and C57BL/6 mice (165.3 ± 13.53, n = 3) did not differ significantly.

Table S1. Sequences of the forward and reverse primers used for RT-qPCR.

Gene	Forward primer	Reverse primer
Gapdh	5′-AGGTCGGTGTGAACGGATTT-3′	5′-TGTAGACCATGTAGTTGAGG-3′
Tslp	5′-ACGGATGGGGCTAACTTACAA-3′	5′-AGTCCTCGATTTGCTCGAACT-3′
Tslpr	5′-CCCACCTTGGAATGTGACG-3′	5′-CCGGAAGTCATAGCAGCGT-3′
Trpv4	5′-ATGGCAGATCCTGGTGATGG-3′	5′-GGAACTTCATACGCAGGTTTG-3′
Trpv3	5′-ACGGTCACCAAGACCTCTC-3′	5′-GACTGTTGGGATTGGATGGGG-3′
Par2	5′-TCCGGTAGAACCAGGCTTTTC-3′	5′-GGGCAAACCAATCACAAACAC-3′
IL-33	5′-TCCAACTCCAAGATTTCCCCG-3′	5′-CATGCAGTAGACATGGCAGAA-3′
IL-13	5′-CCTGGCTCTTGCTTGCCTT-3′	5′-GGTCTTGTGTGATGTTGCTCA-3′
